# Supplementary material for: The Systems Biology Research Tool: evolvable open-source software
Source: BMC Syst Biol. 2008 Jun 29;2:55. doi: 10.1186/1752-0509-2-55 (PMC2446383; doi:10.1186/1752-0509-2-55)
Supplement: Additional file 1 — SBRT Archive. An archive of the current version of the Systems Biology Research Tool. [file 1752-0509-2-55-S1.zip › sbrt-1.4.0/doc/developers_guide/api/sbrt/shell/text/package-frame.html]

sbrt.shell.text


sbrt.shell.text

Interfaces 
  
*AppInputFileLineFormat*
  
*BooleanFormat*
  
*CollectionFormat*
  
*DoubleFormat*
  
*Format*
  
*Formatter*
  
*IntegerFormat*
  
*LinkedHashSetFormat*
  
*ListFormat*
  
*LongFormat*
  
*MapEntryFormat*
  
*MapFormat*
  
*Parser*
  
*PercentFormat*
  
*SimpleFormat*

Classes 
  
AbstractLinkedHashSetFormat
  
AbstractListFormat
  
AbstractMapEntryFormat
  
AbstractMapFormat
  
AmpersandSetFormat
  
AppInputFileLineFormatVersion1
  
BoolRelFormatV1
  
ColonMapEntryFormatV1
  
ColonMapFormatV1
  
DoubleFormatV1
  
DoubleFormatV2
  
EqualsMapEntryFormatV1
  
EqualsMapFormatV1
  
FileFormatFormat
  
IntegerFormatV1
  
LongFormatV1
  
NonEmptyStringFormat
  
PercentFormatVersion1
  
PipeListFormat
  
PipeSetFormat
  
SimpleListFormat
  
SimpleSetFormat
  
SpecialChars
  
StringFormat
  
TabListFormat
  
TabSetFormat
  
TrueFalseFormat
  
WhitespaceListFormat
  
WhitespaceSetFormat
  
YesNoFormat

Exceptions 
  
FormatException
